# The Role of Mental Health Stigma in University Students’ Satisfaction With Web-Based Stress Management Resources: Intervention Study

**DOI:** 10.2196/50018

**Published:** 2024-04-04

**Authors:** Sohyun Cho, Laurianne Bastien, Julia Petrovic, Bilun Naz Böke, Nancy L Heath

**Affiliations:** 1 Department of Educational and Counselling Psychology McGill University Montreal, QC Canada

**Keywords:** help-seeking behavior, help-seeking, mental health services, mental health stigma, mental health, university students, web-based workshop

## Abstract

**Background:**

University students frequently report elevated levels of stress and mental health difficulties. Thus, the need to build coping capacity on university campuses has been highlighted as critical to mitigating the negative effects of prolonged stress and distress among students. Since the COVID-19 pandemic, web-based stress management resources such as infographics and web-based workshops have been central to supporting university students’ mental health and well-being. However, there is a lack of research on students’ satisfaction with and uptake of these approaches. Furthermore, mental health stigma has been suggested to have not only fueled the emergence of these web-based approaches to stress management but may also influence students’ help-seeking behaviors and their satisfaction with and uptake of these resources.

**Objective:**

This study explored potential differences in students’ satisfaction and strategy use in response to an interactive infographic (an emerging resource delivery modality) presenting stress management strategies and a web-based workshop (a more common modality) presenting identical strategies. This study also examined the relative contribution of students’ strategy use and family-based mental health stigma in predicting their sustained satisfaction with the 2 web-based stress management approaches.

**Methods:**

University students (N=113; mean age 20.93, SD 1.53 years; 100/113, 88.5% women) completed our web-based self-report measure of family-based mental health stigma at baseline and were randomly assigned to either independently review an interactive infographic (n=60) or attend a synchronous web-based workshop (n=53). All participants reported their satisfaction with their assigned modality at postintervention (T1) and follow-up (T2) and their strategy use at T2.

**Results:**

Interestingly, a 2-way mixed ANOVA revealed no significant group × time interaction or main effect of group on satisfaction. However, there was a significant decrease in satisfaction from T1 to T2, despite relatively high levels of satisfaction being reported at both time points. In addition, a 1-way ANOVA revealed no significant difference in strategy use between groups. Results from a hierarchical multiple regression revealed that students’ strategy use positively predicted T2 satisfaction in both groups. However, only in the web-based workshop group did family-based mental health stigma predict T2 satisfaction over and above strategy use.

**Conclusions:**

While both approaches were highly satisfactory over time, findings highlight the potential utility of interactive infographics since they are less resource-intensive than web-based workshops and students’ satisfaction with them is not impacted by family-based mental health stigma. Moreover, although numerous intervention studies measure satisfaction at a single time point, this study highlights the need for tracking satisfaction over time following intervention delivery. These findings have implications for student service units in the higher education context, emphasizing the need to consider student perceptions of family-based mental health stigma and preferences regarding delivery format when designing programming aimed at bolstering students’ coping capacity.

## Introduction

### Overview

The overwhelming reports of stress, distress, and mental health difficulties among university students necessitate urgent action to build coping capacity on university campuses. In a recent survey conducted with 96,489 students across 137 universities by the American College Health Association, 51.2% of university students reported moderate psychological distress, while 24% indicated serious psychological distress [1]. As such, building coping capacity on university campuses is critical, given the negative effects of prolonged stress and distress on academic performance, well-being, and daily functioning [2-4].

Recently proposed theoretical models provide insight into the ways in which university students cope with stress and distress. The Health Theory of Coping [5] posits that university students’ approaches to coping with distress and difficulty exist along a continuum ranging from low- to high-intensity strategies that can be classified as healthy (low risk for adverse health outcomes) or unhealthy (high risk for adverse health outcomes). For example, high-intensity unhealthy coping strategies that pose a risk for unintended negative physical, psychological, and social consequences include substance abuse, self-harm, and suicidality. By contrast, high-intensity coping strategies classified as healthy include social support and professional support. Similarly, low-intensity, unhealthy coping strategies include negative self-talk and rumination, while low-intensity, healthy coping strategies include self-soothing and relaxing or distracting activities. This theory further suggests that individuals will move from lower-intensity coping practices (eg, negative self-talk and self-soothing) to higher-intensity practices (eg, suicidal ideation and professional support) proportional to the degree of distress they experience. Regularly engaging in lower-intensity healthy coping behaviors such as self-soothing, positive self-talk, and breathing exercises can enhance one’s future capacity to cope with distress, difficulty, and uncomfortable emotions [5,6].

Previous research has identified lower-intensity healthy coping strategies (eg, progressive muscle relaxation, diaphragmatic breathing, and meditation) that are effective at promoting resilience by reducing distress and increasing well-being in university students [7,8]. Specific cognitive, behavioral, and mindfulness-based approaches have been shown to effectively reduce levels of anxiety, depression, and the physiological stress response [9-11]. These findings emphasize the value of increased availability of lower-intensity resources that promote coping capacity among university students. Additionally, the use of lower-intensity resources to build coping capacity can also decrease the need for more intensive one-on-one therapeutic services, as university mental health services are struggling to meet elevated demands [12-14]. Therefore, providing students with resources and instruction on these accessible, acceptable, and lower-intensity healthy coping strategies is a timely priority for universities [13,14].

### Barriers to Building Coping Capacity in University Students

Barriers to optimal mental health and well-being on university campuses extend beyond a lack of resources [14]. In particular, mental health–related stigma among students functions as a barrier in this context [15,16]. Specifically, mental health stigma has been shown to inhibit help-seeking behaviors among university students [17,18]. According to a systematic review of quantitative and qualitative studies conducted by Clement et al [19], mental health stigma exhibited a negative association with help-seeking. Furthermore, young adults’ perceived stigma from others (eg, family members) regarding seeking mental health treatment undermines their willingness and opportunities to seek help [20]. Similarly, a recent systematic review found that the second most commonly reported barrier by university students to help-seeking behavior was mental health stigma [21]. Specifically, students expressed being worried about their family or friends not being able to understand their situation or that they would perceive them in an unfavorable light.

A body of literature also demonstrates that university students’ cultural, racial, or ethnic identities may have an impact on their help-seeking behaviors. Specifically, university students from certain cultures, races, and ethnic groups may be at greater risk of experiencing mental health difficulties [22] and may be less likely to receive mental health treatment [23]. However, a systematic review and meta-analysis conducted by Wang et al [24] found that culture was not a significant contributor to mental health information-seeking behavior. Thus, the intersectionality of cultural, racial, and ethnic identities in the context of mental health resource use is highly complex, with inconclusive findings, and beyond the scope of this study. Interestingly, as reported in a systemic review by Lui et al [21], stigma around mental health was a key contributor to help-seeking for university students. As such, an individual’s perceptions around the degree of mental health stigma that is present in their familial context provide an opportunity to examine the potential role of mental health stigma in influencing university students’ satisfaction with and use of resources for their mental health and stress management.

Taken together, the above literature demonstrates that there is a need for innovative approaches to the delivery of mental health and well-being resources to university students, which accounts for the impact of mental health stigma on help-seeking in the higher education context. Stigma-related barriers to help-seeking behaviors on university campuses have, in part, fueled the emergence of alternative web-based approaches for sharing stress management and well-being resources with students.

### Emergence and Utility of Web-Based Resources for Stress Management

During the COVID-19 pandemic, web-based resources became central in providing students with stress management support remotely [25] and have been disseminated using different modalities. Particularly, standard in-person workshops were adapted to a web-based format to be delivered on the web. These web-based workshops have gained popularity in the university context, as they allow students to learn and engage with stress management material remotely while still providing them with the opportunity to ask for clarifications from an experienced facilitator. Similarly, mental health and well-being service delivery through interactive infographics has gained popularity in recent years, given their utility for conveying complex information in a concise, visually appealing, and layperson-friendly manner to general audiences [26,27]. Moreover, interactive infographics include features for users to engage with the content (eg, links to guided audio recordings and strategy practice). However, despite their emerging use, research on the use of infographics for promoting mental health and stress management among university students is scarce. Specifically, although user satisfaction and uptake have been highlighted as crucial elements associated with intervention effectiveness in university students [28], to our knowledge, studies have yet to examine students’ acceptability of emerging modalities of resource provision (eg, web-based workshops and interactive infographics). Thus, there is a need for further evidence on students’ satisfaction, uptake, and use of these web-based resources, as well as which factors contribute to satisfaction with these web-based approaches to university mental health resource provision.

Furthermore, investigations into university students’ satisfaction with interactive infographics and web-based workshops for stress management should consider common barriers to students accessing mental health support [15,16]. Given the level of autonomy that is inherent in using web-based mental health resources [29], ensuring adherence to intervention recommendations (eg, at-home practice of strategies taught) is another challenge that may impact web-based resource satisfaction [30,31]. Furthermore, adherence to resources may vary as a function of the modality of web-based stress management resource provision [31,32]. For instance, some self-guided web-based resources (eg, websites) have been associated with lower levels of adherence [32]. Importantly, research has suggested a potential positive association between adherence, which is measured as participants’ frequency of strategy use, and satisfaction with a web-based mental health intervention [33,34]. Thus, there has been a call to monitor and promote adherence (ie, strategy use) in the dissemination of self-guided web-based resources to optimally support user satisfaction with them [35].

### This Study

Drawing on the interdisciplinary literature reviewed above, the overarching aim of this study was to explore students’ relative satisfaction and strategy use when presented with a stress management resource delivered through a web-based workshop (a relatively common mode of resource delivery) versus an interactive infographic (an innovative, emerging mode of resource delivery). This study also aimed to explore potential contributing factors to sustained satisfaction with these 2 modalities of resource delivery, specifically the role of strategy use (ie, adherence) and family-based mental health stigma. The first objective was to examine whether university students’ satisfaction with web-based stress management resources would differ as a function of their delivery format (ie, interactive infographics and web-based workshops) and over time. The second objective was to examine whether students’ strategy use would differ as a function of delivery format. The third objective was to explore the relative contribution of students’ strategy use and family-based mental health stigma in predicting their sustained satisfaction with the interactive infographic versus the web-based workshop. Given the exploratory nature of the study objectives, no specific hypotheses were made. Specifically, for the first objective, based on anecdotal and clinical experience in mental health service delivery, it was anticipated that there might be a differential response such that the participants in the interactive infographic group maintain their satisfaction for a longer period of time than participants in the web-based workshop group given the continued ease of accessing the interactive infographic (ie, self-guided and can be accessed anywhere at any time). However, in the absence of a body of literature examining university students’ satisfaction with the 2 modalities over time, no specific hypothesis was proposed.

## Methods

### Ethical Considerations

This study was approved by the McGill University Research Ethics Board (#21-10-040). Participants who indicated an interest in participating in this study were invited to complete a web-based survey where the first page of the survey was the consent form explaining that participation in this study was optional and completely voluntary, that responses would be confidential, and that participants could choose not to answer any of the questions should they not want to. Each participant was identified on Qualtrics (Silver Lake) using a unique participant ID number associated with their email address; identifiable data (ie, email addresses) were deleted from Qualtrics once data collection was complete. The master list matching participant information to their unique study ID was protected with a password, saved on a password-protected computer, and was only accessible to the principal investigator and graduate student research assistants working on this study. For the web-based workshop, participants were informed ahead of time that they may choose to keep their cameras off for the duration of the workshop and can log in with only their first name or pseudonym to further preserve confidentiality. Participants were compensated CAD $10 (US $7.43) through electronic transfer for each satisfaction survey completed, up to a maximum of CAD $20 (US $14.85) for completing both satisfaction surveys (T1 and T2).

### Participants

A total of 168 undergraduate students were recruited during the Winter 2022 semester. Of those 168 students, 3 graduate students were excluded from the analyses, given evidence suggesting that undergraduate and graduate students have different stressors and coping strategies [36]. In addition, 1 student was excluded for not completing the demographic questionnaire. A total of 164 students were randomized to either the interactive infographic group (n=81) or the web-based workshop group (n=83). After the randomization, 51 were excluded from the primary analyses due to attrition. Thus, the final sample was composed of 113 students (interactive infographic group n=60; web-based workshop group n=53). Of this final sample of 113 students (mean age 20.93, SD 1.53 years), 88.5% (100/113) self-identified as women, 9.7% (11/113) self-identified as men, and 1.8% (2/113) self-identified as nonbinary. The participants self-identified as White (47/113, 41.6%), Asian (44/113, 38.9%), multiple ethnicities (10/113, 8.9%), Arab or Middle Eastern (5/113, 4.4%), Black or African (4/113, 3.5%), and Hispanic or Latinx (3/113, 2.7%). The participants enrolled in diverse faculties, including arts (66/113, 58.4%), science (20/113, 17.7%), dual majors (11/113, 9.7%), nursing (6/113, 5.3%), law (3/113, 2.7%), and others (7/113, 6.2%).

### Intervention Development and Description

Both interventions (ie, the interactive infographic and web-based workshop) were researcher-developed for the purposes of this study and focused on four main areas of stress management: (1) pause or break, (2) positive awareness, (3) kindness to self, and (4) social connection. These areas came from a review of the literature on stress management programs for university students [7,9,37]. Pause or break draws on principles of mindfulness, which may be defined as paying attention to what we sense or experience in this moment, on purpose, and with nonjudgmental acceptance [38,39]. Positive awareness promotes our ability to notice the positive things that happen to us [40-42]. Kindness to self draws on research in the area of self-compassion [43,44]. Lastly, building social connections draws on research in the area of social connectedness, such that the aim is to enhance an individual’s sense of belongingness with other people, groups, or their communities, as well as to maintain and strengthen these connections over time [45-47].

Both the interactive infographic and web-based workshop included psychoeducation around stress as well as evidence-based, low-intensity healthy strategies for stress management and to build coping capacity [5,7,8]. Specifically, students were provided with clear descriptions of each of the 4 areas of stress management described above, as well as a variety of research-based stress management strategies pertaining to each area using clear text descriptions, images, videos, guided audio recordings, links to relevant websites, and podcasts. The interactive infographic and the web-based workshop (including a resource sheet that was provided to all workshop attendees) contained identical content; only the delivery format differed. Table S1 in Multimedia Appendix 1 provides a detailed outline of the intervention content.

### Procedure

#### Overview

Participants were recruited on the web through flyers advertising the study. Potential participants were invited to provide consent and complete a brief demographic survey hosted on the web on Qualtrics. Participants were then randomly assigned to either the interactive infographic group or the web-based workshop group. All participants then received an email providing further information about the study procedure based on their assigned condition and were asked to select their preferred time slot (among 3 choices within the same week) to review the interactive infographic or attend the web-based workshop. Participants were not informed of the nature of the condition to which they were not assigned.

Both the interactive infographic and web-based workshop sessions were scheduled to be delivered at least 1 week after the completion of the demographics survey. After their respective interventions, participants completed a web-based satisfaction questionnaire at 2 time points: immediately after the intervention (postintervention: T1) and 2 weeks later to assess their sustained satisfaction and frequency of strategy use over the 2-week period (follow-up: T2). Between T1 and T2, participants were not provided with any specific instructions or guidelines for practicing strategies. Following completion of the study, participants were debriefed regarding the study purpose and design and received all materials from both interventions.

#### Interactive Infographic Group

Participants in the interactive infographic group received access to the interactive infographic (in PDF format) through Qualtrics. Participants were instructed to review the interactive infographic content and practice the embedded stress management strategies for a total of 30 minutes. To guarantee that participants engaged with the interactive infographic for the full duration, a timer embedded in the Qualtrics page only allowed participants to proceed to the next page of the survey once the 30 minutes had passed. To support participants’ continued use of the strategies presented on the interactive infographic between T1 and T2, the interactive infographic was subsequently shared with participants through email.

#### Web-Based Workshop Group

Participants in the web-based workshop group attended a 30-minute workshop through Zoom (Zoom Video Communications). All workshops were delivered by the same research assistant, who followed an oral script. Similar to the interactive infographic group, to support participants’ continued use of the strategies presented during the web-based workshop between T1 and T2, a 1-page resource sheet summarizing the information and strategies presented during the workshop was shared with participants through email.

### Measures

#### Satisfaction

Participants’ satisfaction with the stress management resources was assessed using an 8-item researcher-developed questionnaire based on the new world Kirkpatrick model for program evaluation [48]. Specifically, the questions assessed participants’ (1) reaction (satisfaction, engagement, and relevance) and (2) learning (knowledge, skills, attitude, confidence, and commitment), which correspond respectively to levels 1 and 2 of the new world Kirkpatrick model. For instance, sample items related to (1) reaction included: “I found the infographic/workshop useful for me and I found that the infographic/workshop was presented in an engaging manner,” while sample items related to (2) learning included:

The strategies presented in the infographic/workshop helped me better understand how to manage my stress and improve my wellness and I feel confident in my understanding of the suggested strategies in the infographic/workshop.

Participants responded on a 4-point Likert scale (1=“strongly disagree” to 4=“strongly agree”). The possible sum satisfaction scores ranged from 8 to 32, where a higher sum score denoted greater satisfaction. This measure demonstrated good internal consistency at T1 (Cronbach α=0.82) and at T2 (Cronbach α=0.90).

#### Strategy Use

Participants’ frequency of strategy use was assessed using a researcher-developed single-item measure at T2. Although participants were introduced to numerous strategies within their stress management resource (Table S1 in Multimedia Appendix 1 provides an outline of the strategies taught), this particular item asks participants about their overall use of any combination of the strategies taught. Specifically, this item corresponds to level 3 (behavior) of the new world Kirkpatrick model for program evaluation [48]. Participants were asked to respond to the following item: “Over the past two weeks, how often did you use the strategies presented in the infographic/workshop?” Participants responded using a 4-point Likert scale (1=“never” to 4=“every day”), where a higher score indicated more frequent use of strategies.

#### Family-Based Mental Health Stigma

Perceived mental health stigma from family members (ie, family-based mental health stigma) was assessed using a researcher-developed single-item measure at baseline. Participants were asked to think about their experiences with mental health-related stigma within their immediate (eg, parents and siblings) or extended family (eg, grandparents, aunts, and uncles) and were asked to respond to the following item: “In my family, I feel there is stigma associated with talking about having mental health difficulties.” Participants responded on a 5-point Likert scale (1=“strongly disagree” to 5=“strongly agree”), where a higher score indicated greater family-based mental health stigma.

### Data Analysis

For the first objective, a 2-way mixed ANOVA was conducted to examine the effect of group (interactive infographic and web-based workshop) and time (T1 and T2) on students’ satisfaction with web-based stress management resources. For the second objective, a 1-way ANOVA was conducted to examine potential group differences in strategy use. Lastly, for the third objective, separate hierarchical multiple regressions were run for the interactive infographic group and the web-based workshop group to examine whether family-based mental health stigma predicted students’ sustained satisfaction with web-based stress management resources at T2, even when controlling for their strategy use.

## Results

### Main Analyses

### Objective 1

The first objective sought to compare group differences in university students’ satisfaction with an interactive infographic versus a web-based workshop for stress management over time (from T1 to T2). A 2-way mixed ANOVA revealed that there was no significant interaction between group (interactive infographic and web-based workshop) and time (T1 and T2) on students’ satisfaction (Table 1). In addition, there was no significant main effect of group, indicating that, regardless of time (T1 and T2), there were no group differences in students’ satisfaction between the interactive infographic and the web-based workshop; overall, students in each group were highly satisfied at both T1 and T2. However, there was a significant main effect of time, where satisfaction decreased for all students from T1 to T2, regardless of their assigned group.

**Table 1:** Results of a 2-way mixed ANOVA (group × time) comparing university students’ satisfaction with 2 stress management resource delivery modalities (N=113).

Time points				Intervention group^a^					Students’ satisfaction^a^			
				Interactive infographic (n=60), mean (SD)		Web-based workshop (n=53), mean (SD)		*F* test *(df)*			η_p_^2^	*P* value
**T1**												
	Interaction			27.67 (2.85)		27.89 (3.23)		0.16 (1,111)			.00	.69
**T2**												
		Main effect of group (between)	25.97 (2.93)		26.40 (3.42)		0.39 (1,111)			.00		.54
		Main effect of time (within)	N/A^b^		N/A		36.81 (1,111)			.25		<.001

^a^The possible range of the postintervention and follow-up satisfaction score was from 8.00 to 32.00.

^b^N/A: not applicable.

### Objective 2

The second objective sought to compare group differences in students’ strategy use with an interactive infographic versus a web-based workshop for stress management over the 2-week study period (assessed retrospectively at T2). A 1-way ANOVA revealed that there was no significant difference in strategy use between the interactive infographic group (mean 2.02, SD 0.57) and the web-based workshop group (mean 2.13, SD 0.62; *F*_4,111_=1.065; *P*=.30).

### Objective 3

The third objective sought to examine the potential contribution of students’ strategy use and family-based mental health stigma in predicting their sustained satisfaction with each web-based stress management approach at T2. We ran 2 hierarchical multiple regression analyses (1 for each group), where strategy use frequency was entered in step 1 and family-based mental health stigma was entered in step 2. Table 2 presents the results of the hierarchical multiple regression analyses.

The hierarchical regression results revealed that, in the infographic group, students’ strategy use frequency explained 25% of the variance in sustained satisfaction at T2 (*F*_1,58_=19.27; *P*<.001; *R*^2^=0.25). Specifically, strategy use frequency (β*=*.50; *P*<.001) emerged as a significant positive predictor of students’ sustained satisfaction at T2. When controlling for strategy use frequency, family-based mental health stigma (β*=*.03; *P*=.83) did not emerge as a significant predictor of sustained satisfaction at T2 (*F*_1,57_=0.05; *P*=.83, Δ*R*^2^=0.00).

In the web-based workshop group, students’ strategy use frequency explained 11% of the variance in sustained satisfaction at T2 (*F*_1,51_=6.14; *P*=.02; *R*^2^=0.11). Specifically, strategy use frequency (β=.33; *P*=.02) emerged as a significant positive predictor of students’ sustained satisfaction at T2. When controlling for strategy use frequency, family-based mental health stigma contributed an additional 10% explained variance in sustained satisfaction at T2 (*F*_1,50_=6.42; *P*=.01; Δ*R*^2^=0.10). Thus, family-based mental health stigma (β=–.32, *P*=.01) emerged as a significant negative predictor of students’ sustained satisfaction at T2. The full model of strategy use frequency and family-based stigma surrounding mental health difficulties significantly predicted sustained satisfaction at T2 in the web-based workshop group (*F*_2,50_=6.60; *P*=.003; *R*^2^=0.21), for a total of 21% explained variance.

**Table 2:** Summary of hierarchical multiple regression for sustained satisfaction at T2 by group.

			Sustained satisfaction at T2															
			Interactive infographic (n=60)									Web-based workshop (n=53)						
			*B*		*SE B*		β		*P* value		*B*			*SE B*		β		*P* value
**Step 1**																		
	Constant	20.76		1.23		N/A^a^		N/A		22.55			1.62		N/A		N/A	
	Strategy use frequency	2.58		0.59		0.50		<.001		1.81			0.73		0.33		.02	
**Step 2**																		
	Constant	20.56		1.54		N/A		N/A		25.04			1.82		N/A		N/A	
	Strategy use frequency	2.59		0.59		0.50		<.001		1.74			0.69		0.32		.02	
	Family-based mental health stigma	0.05		0.25		0.03		.83		–0.75			0.30		–0.32		.01	

^a^N/A: not applicable.

## Discussion

### Overview

Previous literature has noted the promising benefits of web-based stress management resources for university students, including their cost-effectiveness and flexibility [49-52]. However, it remains unclear how readily students accept interactive infographics, a relatively new and innovative resource modality, compared to the widely adopted standard web-based workshops. This study was thus guided by 3 overarching objectives. The first objective was to compare university students’ satisfaction with an interactive infographic versus a web-based workshop over time (from T1 to T2). The second objective was to compare strategy use between students who engaged with an interactive infographic versus a web-based workshop. Building on the first and second objectives, the third objective was to examine whether students’ sustained satisfaction with each of the web-based stress management approaches at T2 could be predicted by their strategy use and family-based mental health stigma.

The findings from this study demonstrated that across time points, university students who received web-based stress management support through the interactive infographic and the web-based workshop did not significantly differ from one another in their satisfaction with the resources. Furthermore, the findings revealed that university students’ strategy use did not significantly differ between the interactive infographic group and the web-based workshop group. Taken together, these findings suggest that when identical psychoeducation content and evidence-based stress management strategies are delivered to university students, their satisfaction and strategy use over time do not differ as a function of whether they received the stress management instruction through an interactive infographic or a web-based workshop. Rather, in this study, students were highly satisfied with both the interactive infographic and the web-based workshop. Since research on the use of interactive infographics for promoting mental health and stress management among university students is scarce, these novel findings suggest that the delivery of stress management support through interactive infographics may be well-received by university students. Thus, the use of interactive infographics within the context of university-wide stress management and mental health programming warrants further investigation, particularly since they are a novel approach and are less resource-intensive than other resources (eg, in-person and web-based workshops). Additionally, as highlighted in the mental health information seeking literature, students are consistently turning to web-based resources for support; thus, there is a need for universities to provide students with new online approaches to resource delivery to enhance their well-being [21]. Offering evidence-based self-directed resources could benefit university students who prefer self-reliance to address mental health difficulties and can mitigate reported challenges such as time constraints and stigma [21,53].

Although many intervention studies measure satisfaction at a single time point [54], the importance of measuring satisfaction over time following intervention delivery has been highlighted, as there is a possibility that acceptability may change during the weeks following exposure to the intervention [55,56]. Indeed, in this study, students’ satisfaction with the interactive infographic and the web-based workshop significantly decreased from T1 to T2. However, it should be noted that, given the small magnitude of these decreases across both groups (ie, less than a 2-point decrease across groups on a scale ranging from 8 to 32), these decreases may not be clinically meaningful. Furthermore, students’ satisfaction remained relatively high across the 2 time points, with mean scores ranging from 25.97 to 27.89 out of 32 for both groups (Table 1). Nevertheless, we propose 3 possible interpretations for the decrease in satisfaction from T1 to T2, even though it remained relatively high over time. First, it is possible that the observed decrease in satisfaction over time was related to students’ initial enthusiasm regarding the strategies presented in the program, which may have been followed by barriers encountered over the two weeks that followed, such as a lack of time to engage in strategy practice [57,58]. This may have, in turn, slightly negatively impacted their satisfaction with the resources. Another potential explanation for this finding is that participants may be less likely to recall resource content with the passage of time, resulting in a less favorable satisfaction rating when followed up with at a later date. Finally, as with any repeated measures design, other unmeasured confounding variables present during the intervention period may explain this time effect as well. Thus, additional studies are needed to examine factors that may influence satisfaction with stress management resources over time to support the sustainability of resource use in the long term.

Furthermore, this study considered that students’ self-directed practice of the strategies presented, along with their family-based mental health stigma, may have an influence on their sustained satisfaction with the interactive infographic and the web-based workshop. Indeed, the frequency of strategy use was a significant predictor of university students’ sustained satisfaction with both modalities at T2. The more frequently participants used strategies over the 2 weeks, the higher they rated their sustained satisfaction with the web-based stress management resource at T2. This finding is not surprising since previous literature on intervention adherence has shown that greater adherence to self-directed web-based interventions is positively related to more favorable outcomes [59]. However, further research into the temporal nature of the relationship between strategy use and satisfaction is needed, as it remains unclear whether increased strategy use leads to greater satisfaction or vice versa. Nevertheless, this finding highlights the degree to which strategy use and sustained satisfaction are intertwined. In the context of stress management intervention design and delivery in university settings, this suggests that importance should be placed on (1) providing students with a variety of strategies such that they can find ones that they like and that work for them, as well as (2) building a community or environment within the university where strategy use is supported and encouraged.

Interestingly, after controlling for the frequency of strategy use, students’ family-based mental health stigma significantly predicted the web-based workshop group’s sustained satisfaction at T2, over and above students’ strategy use. Specifically, when students had greater family-based mental health stigma, their sustained satisfaction with the web-based workshop at T2 was lower. Although it was delivered remotely, the web-based workshop was conducted over Zoom in the presence of a live facilitator and other student attendees, unlike in the context of reviewing an interactive infographic. According to a systematic review conducted by Pretorius et al [29], young people (aged 25 years or younger) tend to be concerned about their privacy and confidentiality even when they are engaging in web-based help-seeking. While participating in the web-based workshop, students with higher levels of family-based stigma may have felt negatively due to the internalization of their perceived stigma from others, as suggested by literature [60,61], which may have negatively influenced their satisfaction. Thus, reports of family-based mental health stigma may have had a greater impact on participants in the web-based workshop group relative to participants in the interactive infographic group because of the relatively lower levels of privacy and confidentiality inherent in workshop participation.

In contrast, students’ family-based mental health stigma did not significantly predict the interactive infographic group’s satisfaction at T2 over and above their strategy use. Due to the relatively private nature of engaging with interactive infographics, students who received the stress management resource through this delivery format did not directly interact with the resource providers or other students while engaging with the infographic. Thus, it is plausible that their satisfaction was less influenced by perceived mental health stigma while acquiring stress management knowledge and practicing the strategies. These findings highlight that, even in the context of web-based stress management initiatives, mental health-related stigma may still be an important factor to consider for university students’ satisfaction with specific resource delivery formats.

### Limitations and Future Directions

While these findings shed new light on university students’ satisfaction with online stress management approaches and the associations between their satisfaction and family-based mental health stigma, they should be interpreted within the context of several limitations. First, this study did not directly address the impact of university students’ culture, race, and ethnicity on their help-seeking behavior and instead focused on students’ perceived family-based mental health stigma. Future studies may wish to explore potential cultural, racial, and ethnic differences in university students’ uptake of and satisfaction with different modalities for mental health resource provision. Second, women accounted for a large majority of the sample (100/113, 88.5%), which also limits the generalizability of findings. Future studies may benefit from examining the acceptability of web-based resources for stress management with samples that are more diverse in terms of gender identity. Third, this study used a researcher-developed single-item approach for measuring strategy use and family-based mental health stigma. A limitation of this approach is that it is impossible to calculate internal consistency estimates of reliability [62]. However, it has been suggested that there is no difference in the predictive validity of single-item measures and multi-item measures [63]. Furthermore, an advantage of using single-item measures is the increased brevity and thus feasibility of capturing a psychological construct (eg, individuals’ beliefs) in a simple screening [62]. The fact that both variables were found to be significantly related to sustained satisfaction, despite being limited to single-item measures, demonstrates that these single-item measures have the potential for use, and it is a first step for tapping into individuals’ perceptions of family-based mental health stigma. Lastly, this study examined students’ satisfaction with the 2 web-based stress management approaches but did not compare their effectiveness. Although this was an important first step, future studies are needed to evaluate the effectiveness of different formats of web-based resource delivery, such as interactive infographics and web-based workshops, on students’ mental health-related outcomes (eg, stress, mindfulness, and well-being). Additionally, given findings on the impact of family-based mental health stigma on satisfaction among students in the web-based workshop group, future studies may want to examine specific strategies to address or mitigate the impact of family-based mental health stigma in the context of web-based workshop delivery.

### Contributions

Despite these limitations, the findings from this study contribute to our understanding of university students’ satisfaction with web-based stress management approaches, in addition to their association with their family-based mental health stigma. Given the impact of stigma on mental health service and resource use [15,64], as well as the paucity of research examining students’ receptivity to interactive infographics for stress management and well-being resource delivery, this study is a first step in demonstrating the relationship between university students perceived mental health stigma and satisfaction with both interactive infographics and web-based workshops. These findings have implications for student service units in the higher education context. Specifically, they highlight the importance of carefully considering student perceptions of mental health stigma and preferences regarding format of delivery when designing programming to support building students’ coping capacity [34,65].

### Conclusion

Since the COVID-19 pandemic, web-based stress management resources are increasingly being delivered to university students to support their elevated mental health and well-being needs. This study provides preliminary evidence that students’ satisfaction with web-based stress management resources may not differ as a function of delivery modality (ie, whether the instruction is delivered through an interactive infographic or a web-based workshop). This study thus has implications for future approaches to mental health service delivery in universities, as the results demonstrate students’ high satisfaction with 2 different web-based delivery formats. Moreover, findings emphasize the negative impact of students’ perceived mental health stigma on their satisfaction with web-based workshops. Overall, while both an interactive infographic and a web-based workshop elicited high levels of satisfaction across time points in this study, results highlight the utility of interactive infographics since they are less resource-intensive than workshops, easy to access and distribute, and given that students’ satisfaction with them is not impacted by family-based mental health stigma.

## Appendix

Multimedia Appendix 1 Table S1. Detailed outline of the intervention content.

## Data Availability

The data generated or analyzed during this study are not publicly available since the authors documented on the institutional ethics board that the data will not be shared with any other researchers.
