# Supplementary material for: The Role of Mental Health Stigma in University Students’ Satisfaction With Web-Based Stress Management Resources: Intervention Study
Source: JMIR Form Res. 2024 Apr 4;8:e50018. doi: 10.2196/50018 (PMC11027058; doi:10.2196/50018)
Supplement: Multimedia Appendix 1 [file formative_v8i1e50018_app1.docx]

| Areas of Stress Management | Psychoeducation Content | Strategies |
| --- | --- | --- |
| Introduction to Stress Management | What is Stress? | N/A |
| Pause/Break | Why Pause/Break?  How to Pause/Break? | **Progressive muscle relaxation**  **Calming breath**  **Coming to your senses**  Hearing/Vision/Taste/Touch/Smell  **Strategies for sleep**  Yoga Nidra meditation  Sleep with me podcast |
| Positive Awareness | Why enhance Positive Awareness? | **Noticing positive moments**  **Three good things**  **Gratitude journal** |
| Kindness to Self | Why Self-Compassion? | **Self-compassion meditation**  **Self-care examples** |
| Social Connection | N/A | **Random acts of kindness**  **Pass it on**  **Getting involved**  Student clubs  Student associations  International student buddy program |
